# Does temporary transfer to preoperative hemodialysis influence postoperative outcomes in patients on peritoneal dialysis? A retrospective cohort study

**DOI:** 10.3389/fsurg.2022.1056908

**Published:** 2023-01-06

**Authors:** Yuyang Zhang, Qingqing Zhou, Zeyang Chen, Jie Dong, Pengyuan Wang

**Affiliations:** ^1^ Department of General Surgery, Peking University First Hospital, Beijing, Republic of China; ^2^ Renal Division, Department of Medicine, Peking University First Hospital, Beijing, Republic of China

**Keywords:** end-stage renal disease, hemodialysis, peritoneal dialysis, surgery, preoperative dialysis, peri-operative management

## Abstract

**Background:**

The associations between preoperative transfer to hemodialysis (HD) and postoperative outcomes in patients on chronic peritoneal dialysis (PD) remain unknown. We conducted this retrospective cohort study to investigate whether preoperative HD could influence surgical outcomes in PD patients undergoing major surgeries.

**Methods:**

All chronic PD patients who underwent major surgeries from January 1, 2007, to December 31, 2020, at Peking University First Hospital were screened. Major surgery was defined as surgical procedures under general, lumbar or epidural anesthesia, with more than an overnight hospital stay. Patients under the age of 18, with a dialysis duration of less than 3 months, and those who underwent renal implantation surgeries and procedures exclusively aimed at placing or removing PD catheters were excluded. Patients involved were divided into either HD or PD group based on their preoperative dialysis status for further analysis.

**Results:**

Of 105 PD patients enrolled, 65 continued PD, and 40 switched to HD preoperatively. Patients with preoperative HD were significantly more likely to develop postoperative hyperkalemia. The total complication rates were numerically higher in patients undergoing preoperative HD. After adjustment, the incidence of postoperative hyperkalemia or any other postoperative complication rates were similar between groups. There were no differences in long-term survival between the two groups.

**Conclusions:**

It does not seem indispensable for PD patients to switch to temporary HD before major surgeries.

## Introduction

End-stage renal disease (ESRD) is a severe disease with a steadily rising prevalence. Globally, the prevalence of patients on kidney replacement therapy was reported to be 759 per million population (pmp) according to the Global Kidney Health Atlas survey (1). Using a large nationwide claims database in China, a recent study reported that the prevalence of dialysis patients has increased from 255.11 pmp in 2013 to 419.39 pmp in 2017, and will continue to grow through 2025 (2). Peritoneal dialysis (PD), a kidney replacement therapy with similar survival data compared to hemodialysis (HD) (3, 4), has gained increasing popularity among ESRD patients. In China, from 2011 to 2019, the number of PD patients increased from 10.4% to 14.0% among the dialysis population (5).

With an increasing number of ESRD patients on long-term PD and their promising prognosis, it is only natural to assume that there will be more and more PD patients pursuing major surgeries, whether for malignancies or benign causes, under elective or emergent situations. Among these special populations, it has been proven by multiple studies that surgical morbidity and mortality are significantly higher, leading to heated discussion concerning peri-operative management to lower these risks (6–8).

Indeed, it has been suggested by some experienced surgeons that the choice of preoperative dialysis modality, among other preoperative strategies, could be of utmost importance (9–11) Previous researches have mainly focused on postoperative dialysis choice. It has been proposed that in PD patients undergoing microinvasive procedures or surgeries with relatively small abdominal incisions, PD can often be resumed within 48 h after surgery with altered ultrafiltration scheme (12, 13). However, it could be beneficial for patients undergoing larger procedures to have a two-to-three weeks delay in resuming PD postoperatively, with or without their PD catheter be removed, depending on whether there are intraabdominal infections (14). Studies concerning preoperative dialysis modality in PD patients are scarce, although intensified PD sessions several days before surgery are recommended. Empirically, in the views of many surgeons, a conversion to HD prior to major surgery could help improve postoperative outcomes for chronic PD patients (10). Compared to patients with PD, levels of preoperative serum creatinine (SCr) and blood urea nitrogen (BUN) on HD patients would reach a minimum after each dialysis session, presenting more satisfactory laboratory results statistically before surgery. Supporting theoretical explanations may exist; nevertheless, based on the principle of evidence-based medicine, we need more clinically derived facts.

Based on these unsolved and underresearched clinical problems, we conducted this retrospective cohort study to explore whether temporary preoperative HD correlates with surgical morbidity and mortality in long-term PD patients undergoing major surgeries.

## Methods

### Population

**Figure 1:**
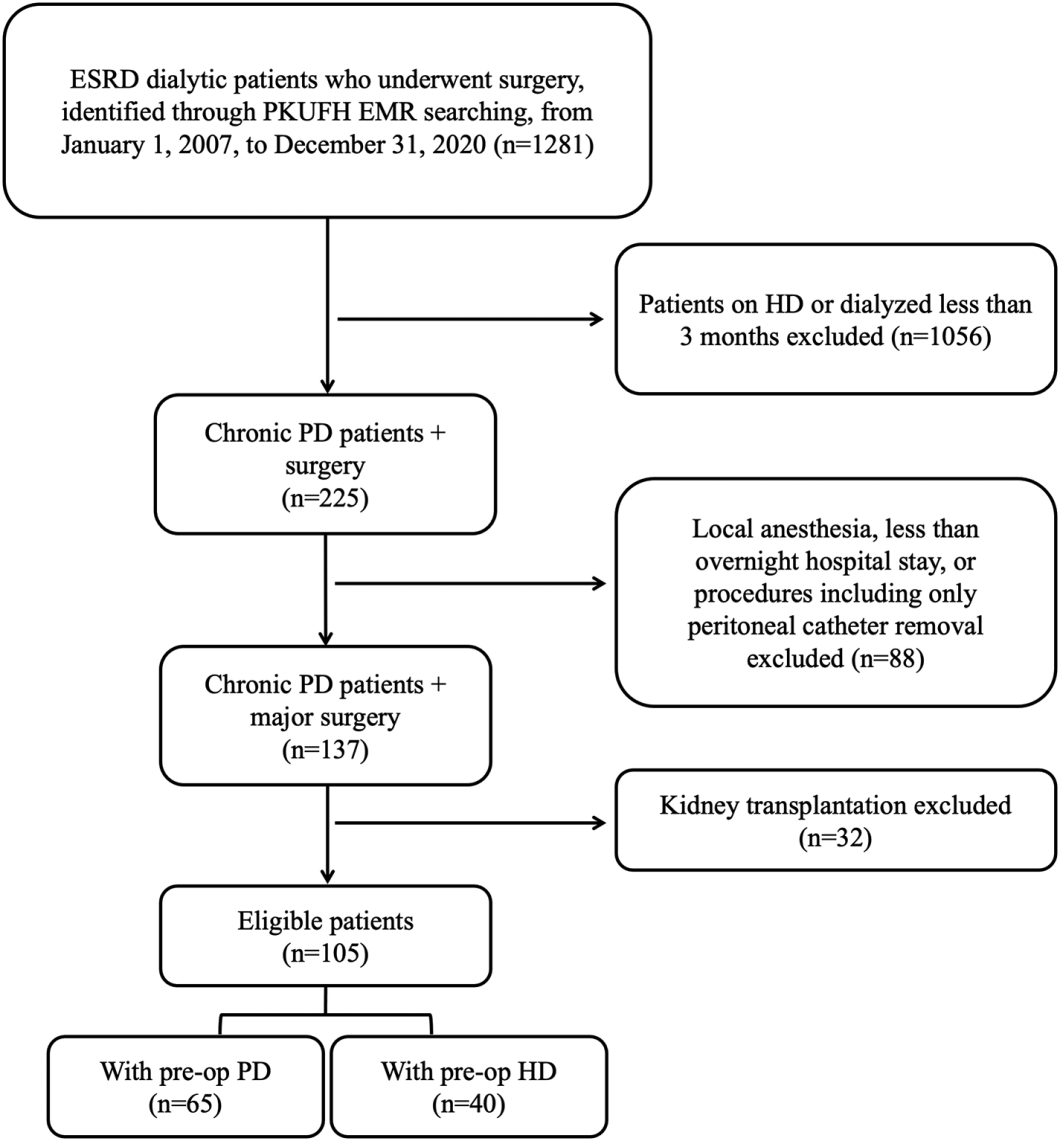
Flow chart of patient selection. ESRD, end-stage renal disease; PKUFH, peking university first hospital; EMR, electronic medical record; PD, peritoneal dialysis; HD, hemodialysis; preop, preoperative.

All ESRD patients on chronic PD who underwent major surgery at Peking University First Hospital (PKUFH) from January 1, 2007, to December 31, 2020, were retrospectively screened from PKUFH electronic medical record (EMR) system. Major surgery was defined as surgical procedures under general, lumbar or epidural anesthesia, with more than an overnight hospital stay. Patients under the age of 18 or with a dialysis duration of less than 3 months were excluded. Renal implantation surgeries and procedures exclusively aimed at placing or removing PD catheters were excluded**.** According to aforementioned inclusion and exclusion criteria, 105 PD patients were included in the study. Patients were divided into either PD or HD group based on their preoperative dialysis status for further analysis. The preoperative PD group included patients who continued with PD prior to surgery, while patients in the preoperative HD group were temporarily transferred to HD preoperatively. The flow chart of patient selection was shown in Figure 1.

### Data collection

Clinical information was obtained mainly from the PKUFH EMR system by an experienced physician and double-checked by another. Baseline demographic and clinical characteristics [including patient age, sex, body mass index (BMI)], dialysis duration, cause of renal failure, and medical history [including diabetes mellitus (DM), cardiovascular disease (CVD), hypertension (HTN), cerebral vascular accident (CVA)/stroke, lung disease, peripheral vascular disease, smoking history, malignancy history, and operative history] were collected. The latest preoperative laboratory data prior to the index surgery were recorded, which included the preoperative hemoglobin level (HGB), BUN level, SCr level, serum albumin, and serum potassium level. Surgical data included the patients' American Society of Anesthesiologists (ASA) score, New York Heart Association (NYHA) classification, wound classification, type of procedure (general, cardiac, urinary, orthopedics, thoracic, burn and plastic, neurosurgical, and gynecological), anesthetic type (general or lumbar/intraspinal), operative approach (open/minimally invasive), elective/emergent procedure, length of procedure, estimated amount of blood loss, and amount of blood transfusion. Postoperative outcomes included postoperative laboratory results, days of hospital stay and days of ICU (intensive care unit) stay, in-hospital mortality, returning to the operation room, postoperative complications, and patient survival. Days of hospital stay were counted from patient admission to discharge. Postoperative laboratory data closest to the index surgery were collected, which included postoperative BUN, SCr, and serum potassium levels. In patients underwent postoperative dialysis, laboratory data were obtained before the first postoperative dialysis session. Postoperative complications were recorded and classified as follows: wound complications (including surgical site infection, hematoma, and wound disruptions), infections (surgical site infection excluded), electrolyte disturbance and acidosis including hyperkalemia (serum potassium level ≥5.5 mmol/L), CVA/stroke, myocardial infarction (MI), cardiac arrhythmia, pulmonary embolism (PE)/deep vein thrombosis (DVT), psychosis, hemorrhage, glucose abnormality (including hyperglycemia and hypoglycemia), and hypotension. Follow-up for postoperative complications ended at the dismission of the patient. Survival data were retrieved either from EMR or from PKUFH peritoneal center records, whichever was more recent, and were renewed through telephone or e-mail in all reachable cases until January 31, 2022. The study was approved by the Institutional Ethics Committee of PKUFH, and patient consent was waived because of the retrospective nature of this article, and no identifiable personal information was recorded.

### Definition of outcomes

The primary outcomes of this study were in-hospital mortality and postoperative complications, as described in the “Data collection” section. Secondary outcomes were postoperative laboratory results, days of hospital stay, days of ICU stay, returning to the operation room, and long-term survival.

### Statistical analysis

Patient data were presented as mean ± standard error (SD) for continuous variables and number (percentage) for categorical variables. We removed missing data by listwise deletion. Variables with >20% missing data were excluded from further analysis. Continuous variables were compared using unpaired t test, while categorical variables were compared using Pearson's *χ*^2^ test or Fisher's exact test. To assess the associations between preoperative dialysis status and postoperative outcomes, the unadjusted hazard ratio (HR) was determined for the preoperative HD group vs. the preoperative PD group using the Cox proportional hazards model. To decide which factors may carry weight on the aforementioned correlation analysis, we conducted univariate analysis using unpaired t test for continuous variables and Pearson's *χ*^2^ test or Fisher's exact test for categorical variables. We selected preoperative or surgical characteristics that were potentially associated with (*p *< 0.1) the two outcomes of particular interest (postoperative serum potassium level and total complications). Six covariates, including preoperative serum potassium level, length of surgical procedure, ASA score, NYHA classification, wound classification, and type of procedure, were incorporated into a Cox proportional hazards regression model to calculate the adjusted HR of postoperative outcomes. The Kaplan‒Meier survival model and log-rank test were used to conduct time-to-event analysis and compare patient survival. All data were analyzed using SPSS Statistics (version 27), and *p* < 0.05 was considered statistically significant.

## Results

### Baseline and surgical characteristics

A total of 105 patients with long-term PD who underwent major surgeries between 2007 and 2020 at PKUFH were included. Forty patients changed the preoperative dialysis modality to HD; 65 continued PD treatment. Baseline patient characteristics, stratified by preoperative dialysis status, are shown in Table 1. Demographic factors (age, sex, and BMI) showed no significant differences between the two groups. There were no significant differences on medical history between two groups. The average dialysis duration was 3.47 (3.36) years for the preoperative PD group and 3.40 (4.31) for the preoperative HD group, without significant differences (*p *= 0.928). The distribution of the main causes of ESRD showed no significant differences between the two groups (*p *= 0.935). Preoperative laboratory results were measured, including HGB, BUN level, SCr level, serum albumin, and serum potassium level. Among these figures, patients from the preoperative PD group had markedly higher preoperative BUN and serum albumin levels (24.02 ± 7.37 mmol/L vs. 17.34 ± 7.85 mmol/L, *p* < 0.001; 35.41 ± 5.19 g/L vs. 32.87 ± 3.66 g/L, *p* = 0.008).

**Table 1:** Patient characteristics by preoperative dialysis Status.

	No. (%)		
Characteristics	Preoperative PD	Preoperative HD	*p* value
Patient number	65	40	
Age, years, mean (SD)	59.26 (12.25)	59.25 (13.59)	0.996
Female, No. (%)	23 (35.4)	15 (37.5)	0.827
BMI, mean (SD)	23.62 (2.86)	24.32 (4.21)	0.311
Medical history, No (%)			
DM	29 (44.6)	19 (47.5)	0.773
CVD	16 (24.6)	8 (20.0)	0.584
HTN	50 (76.9)	34 (85.0)	0.315
CVA/stroke	8 (12.3)	4 (10.0)	0.964
Lung disease	6 (9.2)	5 (12.5)	0.839
Peripheral vascular disease	8 (12.3)	4 (10.0)	0.964
Malignancy history	4 (6.2)	3 (7.5)	1.000
Operative history	39 (60.0)	22 (55.0)	0.614
Smoking history, No. (%)	25 (38.5)	11 (27.5)	0.250
Dialysis duration, years, mean (SD)	3.47 (3.36)	3.40 (4.31)	0.928
Cause of renal failure, No. (%)			0.895
DM	18 (27.7)	11 (27.5)	
HTN	7 (10.8)	6 (15.0)	
Chronic interstitial nephritis	7 (10.8)	4 (10.0)	
Chronic glomerulonephritis	5 (7.7)	4 (10.0)	
Miscellaneous	7 (10.8)	2 (5.0)	
Unknown	21 (32.3)	13 (32.5)	
Preoperative laboratory results, mean (SD)			
HGB (g/L)	106.26 (17.81)	103.3 (13.87)	0.372
BUN (mmol/L)	24.02 (7.37)^a^	17.34 (7.85)	<0.001
SCr (μmol/L)	862.67 (261.80)^b^	928.34 (1276.48)	0.693
Albumin (g/L)	35.41 (5.19)^c^	32.87 (3.66)	0.008
K^+^ (mmol/L)	4.27 (0.66)^d^	4.44 (0.69)	0.239

PD, peritoneal dialysis; HD, hemodialysis; SD, standard error; DM, diabetes mellitus; CVD, cardiovascular disease; HTN, hypertension; CVA, cerebral vascular accident; HGB, hemoglobin; BUN, blood urea nitrogen; SCr, serum creatinine; K^+^, serum potassium level.

^a^ Data missing in 2 patients.

^b^ Data missing in 2 patients.

^c^ Data missing in 1 patient.

^d^ Data missing in 1 patient.

Surgery profiles are depicted in Table 2. In both groups, the procedures conducted were mostly comprised of general, urinary and cardiac surgeries, and the majority of them were elective surgeries. General procedures included in this study mainly consisted of hernia repairs, urinary procedures were predominantly made up of radical resection of urinary tumors and parathyroidectomy, and all cardiac procedures were CABG surgeries. ASA and NYHA status were similar between groups. There were significantly more type I incisions in the preoperative PD group (80.0% vs. 52.5%, *p *= 0.003). There were 30.8% and 17.5% lumbar/intraspinal anesthesia in the preoperative PD and preoperative HD group respectively (*p *= 0.131). More laparoscopic procedures were performed in the preoperative HD group, and the average length of the procedure was also longer than that in the preoperative PD group (12.5% vs. 1.5%, *p *= 0.029; 2.45 ± 1.54 h vs. 1.81 ± 1.14 h, *p *= 0.028). Estimated blood loss and blood transfusion were similar between groups.

**Table 2:** Surgical profile by preoperative dialysis status.

	No. (%)		
Surgery Profile	Preoperative PD	Preoperative HD	*p* value
Type of procedure			0.230
General	39 (60.0)	17 (42.5)	
Cardiac	6 (9.2)	6 (15.0)	
Urinary	6 (9.2)	8 (20.0)	
Orthopedics	6 (9.2)	2 (5.0)	
Thoracic	1 (1.5)	5 (12.5)	
Burn and plastic	4 (6.2)	0	
Neurosurgical	2 (3.1)	1 (2.5)	
Gynecological	1 (1.5)	1 (2.5)	
ASA score			0.738
3	58 (89.2)	34 (85.0)	
>3	7 (10.8)	6 (15.0)	
Anesthetic type			0.131
General	45 (69.2)	33 (82.5)	
Lumbar/intraspinal	20 (30.8)	7 (17.5)	
NYHA classification			0.993
I	33 (50.8)	20 (50.0)	
II	22 (33.8)	14 (35.0)	
III	7 (10.8)	6 (15.0)	
IV	3 (4.6)	0	
Wound classification			0.003
I	52 (80.0)	21 (52.5)	
II	10 (15.4)	19 (47.5)	
III	3 (4.6)	0	
Operative approach			0.029
Open	59 (90.8)	30 (75.0)	
Laparoscopic	1 (1.5)	5 (12.5)	
VATS	1 (1.5)	4 (10.0)	
Cystoscopic	3 (4.6)	1 (2.5)	
Hysteroscopic	1 (1.5)	0	
Elective/emergent			0.347
Elective	59 (90.8)	39 (97.5)	
Emergent	6 (9.2)	1 (2.5)	
Length of procedure, hours, mean (SD)	1.81 (1.14)	2.45 (1.54)	0.028
Estimated blood loss, ml, mean (SD)	140.55 (296.01)^a^	176.14 (214.06)^b^	0.534
Blood transfusion, ml, mean (SD)	84.62 (228.62)	57.50 (143.02)	0.503

PD, peritoneal dialysis; HD, hemodialysis; ASA, american society of anesthesiologists; NYHA, new york heart association; VATS, video-assisted thoracoscopic surgery; SD, standard error.

^a^ Data missing in 3 patients.

^b^ Data missing in 5 patients.

### Associations of postoperative outcomes with preoperative dialysis modality

Postoperative outcomes between the preoperative PD and preoperative HD groups are provided in Table 3. With regard to the primary outcomes, patients in the preoperative HD group had a markedly greater risk of postoperative hyperkalemia (42.5% vs. 21.5%, *p *= 0.022). Additionally, the incidence of total complications was also significantly higher in the preoperative HD group (55.0% vs. 35.4%, *p *= 0.049). Nonetheless, in-hospital mortality was similar between the two groups (*p* = 0.347). For the secondary outcomes, compared with the preoperative HD group, patients with preoperative PD had a significantly higher postoperative BUN and SCr level as above but a markedly lower postoperative serum potassium level (4.64 ± 0.72 mmol/L vs. 5.15 ± 0.82 mmol/L, *p *= 0.002). There were no significant differences in the chances of returning to the operating room, days of hospital stay, or days of ICU stay.

**Table 3:** Postoperative outcomes for PD patients by preoperative dialysis status.

Postoperative outcomes	Preoperative PD	Preoperative HD	*p* value
Postoperative laboratory variables, mean (SD)			
BUN (mmol/L)	26.10 (8.71)^a^	18.88 (7.02)^b^	<0.001
SCr (μmol/L)	885.73 (310.78)^c^	761.24 (244.44)^d^	0.042
K^+^ (mmol/L)	4.64 (0.72)^e^	5.15 (0.82)^f^	0.002
Days of hospital stay, mean (SD)	11.88 (13.48)	12.88 (17.34)	0.742
Days of ICU stay, mean (SD)	1.80 (3.81)	1.83 (2.25)	0.970
Return to operation room, No. (%)	1 (1.5)	0	1.000
Postoperative complications, No. (%)			
Total	23 (35.4)	22 (55.0)	0.049
Hyperkalemia	14 (21.5)	17 (42.5)	0.022
Infection	10 (15.4)	4 (10.0)	0.431
Hemorrhage	6 (9.2)	2 (5.0)	0.678
Hypotension	6 (9.2)	2 (5.0)	0.678
Others	9 (13.8)	4 (10.0)	0.783
Glucose abnormality	4 (6.2)	0	
Cardiac arrhythmia	3 (4.6)	3 (7.5)	
MI	2 (3.1)	0	
Wound	2 (3.1)	0	
Stroke/CVA	1 (1.5)	0	
PE/DVT	1 (1.5)	0	
Psychosis	0	1 (2.5)	
In-hospital mortality, No. (%)	6 (9.2)	1 (2.5)	0.347

PD, peritoneal dialysis; HD, hemodialysis; SD, standard error; BUN, blood urea nitrogen; SCr, serum creatinine; K^+^, serum potassium level; ICU, intensive care unit; MI, myocardial infarction; CVA, cerebral vascular accident; PE/DVT, pulmonary embolism/deep vein thrombosis.

^a^ Data missing in 11 patients.

^b^ Data missing in 1 patient.

^c^ Data missing in 11 patients.

^d^ Data missing in 1 patient.

^e^ Data missing in 11 patients.

^f^ Data missing in 1 patient.

**Figure 2:**
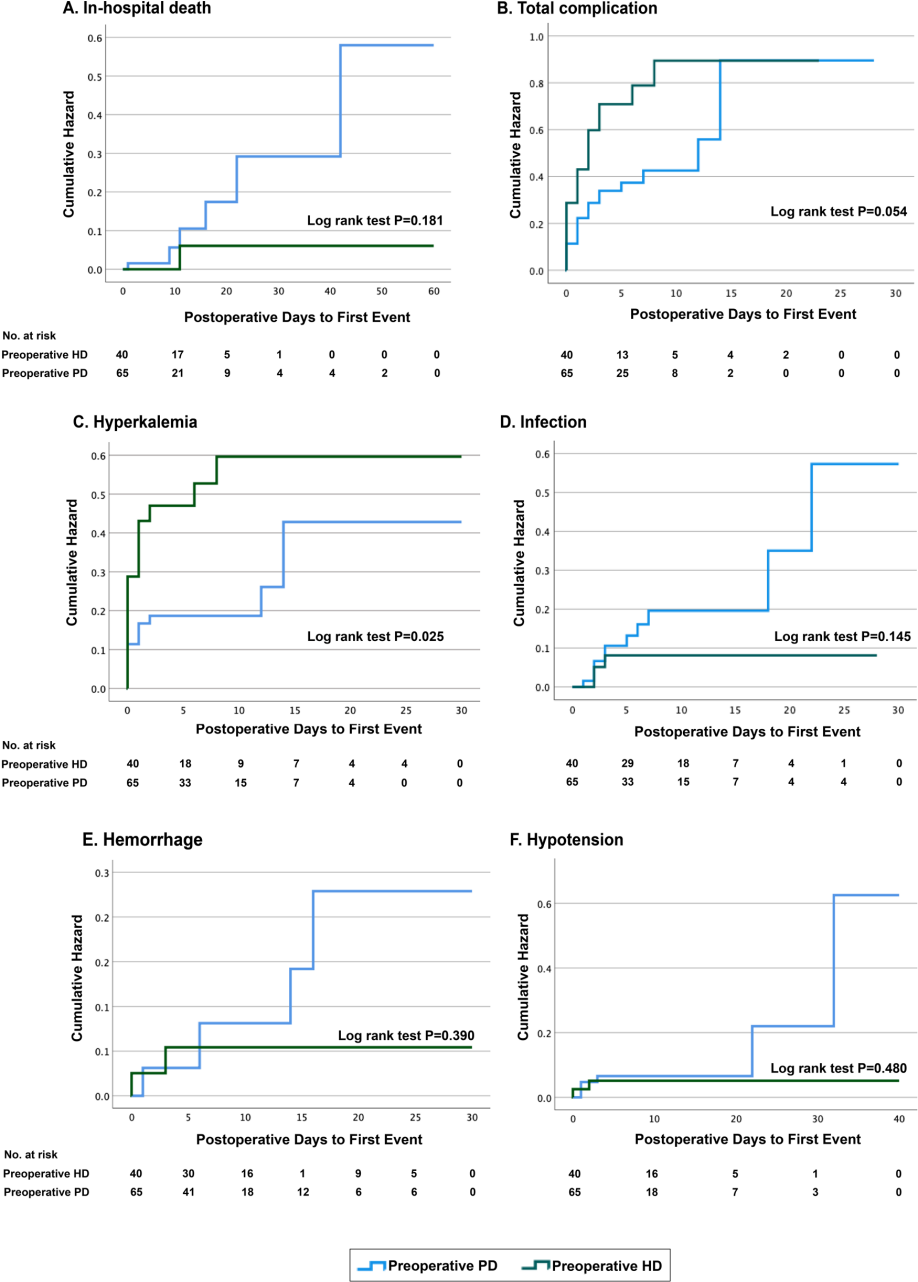
Kaplan–Meier time-to-event analysis of postoperative outcomes. PD, peritoneal dialysis; HD, hemodialysis.

Time-to-event analyses of the two primary outcomes using Kaplan‒Meier estimates are shown in Figure 2. We observed a significantly earlier onset of postoperative hyperkalemia in the preoperative HD group (*p* = 0.025). In accordance with the comparison in Table 3, the likelihood of in-hospital death or any other postoperative complications was similar between the preoperative PD and preoperative HD groups.

**Figure 3:**
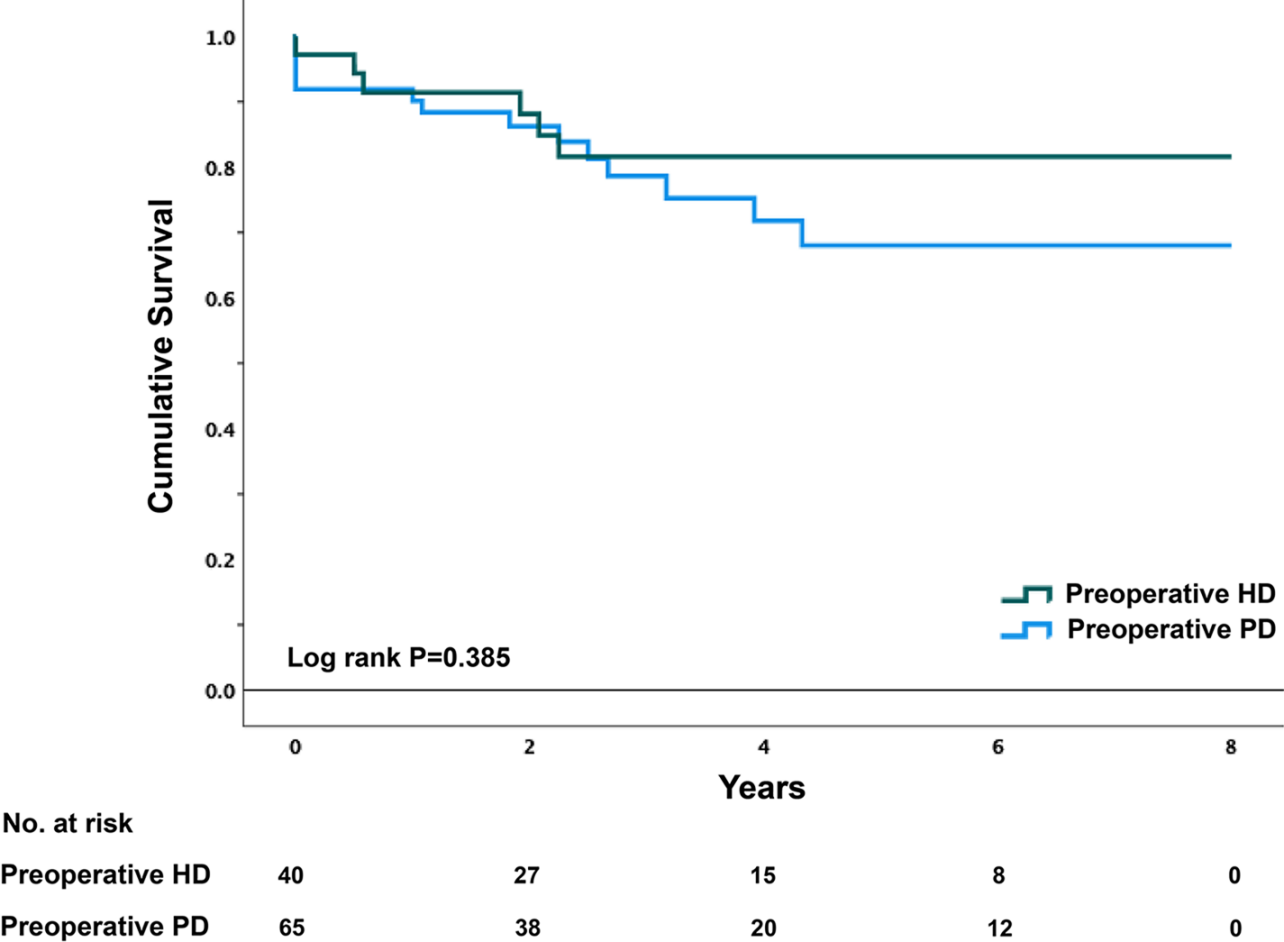
Kaplan–Meier survival plot of PD patients by preoperative dialysis Status. PD, peritoneal dialysis; HD, hemodialysis.

The unadjusted and adjusted HRs of the primary outcomes are presented in Table 4. Patients with preoperative HD were significantly more likely to develop postoperative hyperkalemia (HR = 2.111, 95% CI, 1.040–4.286, *p* = 0.039). However, total complication rates were only numerically higher in patients undergoing preoperative HD (HR = 1.711; 95% CI, 0.953–3.072, *p *= 0.072). After adjusting for preoperative serum potassium level, length of surgical procedure, ASA score, NYHA classification, wound classification, and procedure types, the incidence of postoperative hyperkalemia (HR = 1.261, 95% CI, 0.570–2.789, *p* = 0.566) or any other postoperative complications was similar between the two groups. In terms of in-hospital mortality, there were no significant differences between the two groups with or without adjustment. The average survival times were 6.765 (0.459) years and 6.114 (0.431) years for the preoperative PD and HD groups, respectively. The long-term cumulative survival rates are illustrated in Figure 3, with no significant differences between the two groups (*p *= 0.385).

**Table 4:** Hazard ratio^a^ of postoperative outcomes for PD patients with preoperative HD.

Postoperative outcomes	Unadjusted HR (95% CI)	*p* value	Adjusted HR^b^ (95% CI)	*p* value
**Postoperative complications**				
Total	1.711 (0.953–3.072)	0.072	1.156 (0.590–2.265)	0.673
Hyperkalemia	2.111 (1.040–4.286)	0.039	1.261 (0.570–2.789)	0.566
Infection	0.402 (0.112–1.444)	0.163	0.360 (0.090–1.432)	0.147
Hemorrhage	0.503 (0.101–2.495)	0.400	0.208 (0.031–1.401)	0.107
Hypotension	0.566 (0.114–2.819)	0.487	0.167 (0.018–1.586)	0.119
In-hospital mortality	0.260 (0.031–2.188)	0.215	0.223 (0.017–2.895)	0.251

PD, peritoneal dialysis; HD, hemodialysis; HR, hazard ratio; CI, confidence interval.

^a^ Hazard ratio is presented in relation to patients continuing preoperative PD.

^b^ Adjusted for preoperative serum potassium level, length of surgical procedure, ASA score, NYHA classification, wound classification, and type of procedure.

## Discussion

To our knowledge, this is the first study investigating the effect of preoperative dialysis modality on postoperative outcomes in long-term PD patients undergoing major surgeries. With complete and detailed recording of data and a rather long follow-up time, we found that although PD patients switching to preoperative HD were more likely to suffer from postoperative complications, particularly hyperkalemia, there were no significant differences between the two groups after adjusting for relative baseline and surgery-related factors. In conclusion, preoperative dialysis type may not have a significant impact on postoperative outcomes in chronic PD patients.

In regard to the perioperative management of dialysis patients, most surgeons are convinced that patients treated with long-term PD undergoing major surgeries should switch to HD, at least temporarily, during the preoperative period (9, 11, 15). Some of the plausible reasons are as follows: (a) HD turns out to be a more ideal and convenient way to control the levels of SCr and BUN, and a lower level of preoperative SCr and BUN is regarded as a predictor for a better postoperative outcome (6, 12); (b) preoperative HD sessons can provide more concise preoperative volume control, which could bring cartiovascular benefits (11); (c) preoperative PD might have the risk of dialysate leakage from abdominal incisions, resulting in delayed wound healing and peritonitis (11, 13, 15). However, all these concerns lack support from real-world clinical data and investigations.

Previous studies have indicated that patients with preoperative PD can tolerate surgeries well without interim HD. Inguinal hernia surgeries and cardiac surgeries were performed successfully on patients continuing PD (16, 17). A Chinese group also reported that 58 patients with PD treatment underwent nonabdominal operations safely with no serious complications (18). However, these studies with small sample sizes, limited procedure types, and lack of comparison for switching to HD were inconclusive. To date, no consensus has been reached on whether patients treated with PD should switch to HD before major surgeries.

Our study fills the gap in this field. Firstly, we observed that patients switching to HD preoperatively had a significantly lower postoperative BUN and SCr levels. This could be explained by a major difference in dialysis principles between PD and HD: HD is more effective in removing small molecules and water while PD is better capable at the removal of middle molecular substances (19). In spite of that, patients in the preoperative HD group seemed to have a higher proportion of postoperative complications than patients in the preoperative PD group, especially a higher incidence of hyperkalemia. For further analysis, we found that patients with preoperative PD tended to have a higher level of preoperative albumin and underwent more open procedures with a relatively shorter duration, which might partially explain the lower incidence of postoperative complications in the PD group (20, 21). In regard to hyperkalemia, we attribute the higher incidence and increased risk in the HD group partly to the difference between the two types of dialysis. Hyperkalemia could happen after HD sessions (22), whereas PD patients more often suffer from hypokalemia as one of the most common metabolic complications because the PD fluid does not contain potassium as a supplement (11). However, after adjusting for factors that are potentially related to postoperative complications (including preoperative SCr, preoperative albumin, preoperative potassium, length of procedure, hypertension, ASA score, type of surgery, NYHA grade of cardiac function, and wound classification), no differences were found in the risk of postoperative complications between the two groups, indicating that preoperative dialysis modality has little or no effect on postoperative outcomes of chronic PD patients. Thus, it does not seem indispensable for PD patients to switch to temporary HD before surgery.

Perioperative management for patients treated with PD has been a topic with numerous misunderstandings. Previous studies have confirmed the feasibility and safety of continuing PD treatment during the perioperative period. Some experts even believe that HD patients should switch to interim PD during the perioperative period to avoid hemodynamic instability and decrease the risk of heparin-associated bleeding (23, 24). In addition to aforementioned disadvantages of HD, there are problems concerning vascular access of hemodialysis, such as catheter-related bacteremia (25), which could lead to adverse postoperative outcomes. Intensifying PD in advance of surgeries as well as resuming small-volume PD in the supine position within 48 h after surgeries might help PD patients avoid interim HD, especially for those undergoing nonabdominal procedures or surgeries with small abdominal incisions (11, 16, 26). Based on our findings and previous investigations, we recommend careful consideration when deciding on preoperative dialysis modality for PD patients. Unless strong evidence on the disadvantages of continuing PD are published, long-term PD patients are not necessarily transferred to HD prior to major surgeries.

Although the dialysis principles of PD and HD are different, adequate dialysis is defined as the effective administration of dialysis, which keeps a patient clinically asymptomatic and active and maintains sufficient correction of the altered metabolic and homeostatic components secondary to the loss of renal function (26). Low SCr and BUN levels *per se* are not determinants for dialysis adequacy, unlike traditional thoughts in many surgeons. The urea kinetic model, which was originally designed for HD prescription, has been applied to PD without sufficient verification. According to this model, patients on chronic PD would be considered underdialyzed. However, compared to HD patients, most PD patients do not have more uremic symptoms (27, 28), worse quality of life or long-term survival (9–11), whether residual kidney function exists or not. When evaluating the preoperative state of dialysis patients, we should not only focus on the values of SCr and BUN on the laboratory test sheet but also on more comprehensive indicators such as anemia correction, volume status, cardiac function, mineral metabolism, nutrition status, and individual psychological status (29, 30). In accordance with these theories and study results, in our study, despite the fact that preoperative PD patients generally had higher SCr and BUN levels comparing to preoperative HD patients, there were no salient differences in postoperative outcomes between the two groups, possibly suggesting a similar preoperative physiological status.

Some limitations in our study must be acknowledged. First, this is a retrospective cohort study with all its inherent drawbacks. Nevertheless, it is actually unfeasible to conduct randomized controlled trials or any interventional studies in these special and delicate groups of patients. Second, although our data collected complete information from the EMR system of the hospital and one of the most advanced PD centers in China, this study could only include 105 eligible patients, which may not have sufficient power to detect some of the significant differences. In addition, we found that most PD patients included in our study underwent several specific types of surgery, especially hernia repair and coronary artery bypass grafting (CABG), resulting in a lack of variety in surgical types. Procedures conducted inside the abdominal cavity with removal of a larger portion of the peritoneum, particularly those pertaining to abdominal malignant tumors, were rarely performed in our study. Therefore, the perioperative management of patients on PD undergoing these surgeries requires further investigation. Finally, the advancement in surgical quality and protocols during the past decade would bring in additional confounding variables.

## Data Availability

The raw data supporting the conclusions of this article will be made available by the authors, without undue reservation.
